# Editorial: Epigenetics of Glucose Homeostasis

**DOI:** 10.3389/fendo.2022.889189

**Published:** 2022-04-22

**Authors:** Senta Georgia, H. Efsun Arda, Aida Martinez-Sanchez, Sangeeta Dhawan

**Affiliations:** ^1^ Children’s Hospital Los Angeles, Keck School of Medicine, University of Southern California, Los Angeles, CA, United States; ^2^ Laboratory of Receptor Biology and Gene Expression, Center for Cancer Research, National Cancer Institute, National Institutes of Health, Bethesda, MD, United States; ^3^ Section of Cell Biology and Functional Genomics, Department of Metabolism, Digestion and Reproduction, Faculty of Medicine, Imperial College London, London, United Kingdom; ^4^ Department of Translational Research and Cellular Therapeutics, Arthur Riggs Diabetes and Metabolism Research Institute, City of Hope, Duarte, CA, United States

**Keywords:** epigenetics, epigenetic inheritance, chromatin, diabetes, metabolism

There has been a dramatic increase in the incidence of diabetes in the past few decades, corresponding to major changes in our lifestyle such as over-nutrition and circadian disruption. Moreover, the genetic variants associated with diabetes that have been identified to date, account for only a small fraction of diabetes cases. This has led to intense efforts in understanding the contribution of epigenetic mechanisms to the regulation of glucose homeostasis. Recent technical advances now enable a single-cell resolution view of cellular epigenomes and have revolutionized our understanding of how the epigenome shapes cellular function. This Research Topic on “Epigenetics of Glucose Homeostasis” offers a collection of review articles that summarize a wide spectrum of advances in understanding the effect of environmental factors on tissue function in health and diabetes.

Pancreatic beta-cells are at the center of systemic glucose homeostasis and serve as body’s glucose sensors. Four reviews cover the epigenetic underpinnings of beta-cell function. Brown and Matveyenko present an overview of how nutrient availability regulates beta-cell epigenome and function, and conclude that chronic nutrient overload is a detriment to beta-cell function and survival. Related to nutrient availability, there is significant recent interest in the health benefits of time restricted feeding. This article covers the effect of feeding-fasting behavior on beta-cell epigenome and function, and makes a compelling case for the role of the timing of nutrient exposure in shaping beta-cell epigenome. The review by Parveen and Dhawan focuses on the epigenetic control of beta-cell homeostasis over the lifespan by DNA methylation. The epigenetic control is disrupted under diabetic conditions; this review summarizes recent advances in the development of epigenetic biomarkers and therapeutics to identify and remedy beta-cell defects. The authors review the mechanistic basis of the high degree of epigenetic vulnerability in embryonic development and its impact on future metabolic fitness, noting that adult beta-cells recapitulate key developmental epigenetic and gene-expression hallmarks in the setting of diabetes. One such important set of genes that is controlled by DNA methylation is the “imprinted genes”, as discussed by Villenueva-Hayes and Millership. Imprinted genes are hallmarked by monoallelic expression determined by the parental origin of alleles, a mechanism designed to provide strict dosage-control for these genes that regulate growth and differentiation. This review summarizes the essential role of imprinted genes in beta-cell function, and elaborates on the impact of nutrition on their regulation, especially in their transgenerational control and its relevance to diabetes risk and pathogenesis. The impact of nutrition on epigenome is further illustrated in the review by Sałówka and Martinez-Sanchez, which elaborates on how nutrients such as glucose and fatty acids contribute to shaping the microRNA profiles in beta-cells. miRNAs are the most well studied non-coding RNA species and well-established regulators of multiple metabolic functions. Their article showcases the importance of these small RNAs in beta-cell homeostasis and covers the latest advances in the molecular mechanisms that mediate nutrient control of beta-cell miRNA landscape.

The significance of non-coding RNAs in glucose homeostasis is complemented by the review by Jacovetti et al., which focuses on several emerging non-coding RNA species such as piRNAs (Piwi interacting RNAs), snoRNAs (small nucleolar RNAs) and tRFs (tRNA derived small RNAs). Both piRNAs and tRFs have been shown to contribute to transgenerational inheritance of metabolic phenotypes, while snoRNAs play a role in redox homeostasis and inflammation induced beta-cell death. The growing interest in these RNAs, combined with advances in transcriptomic profiling, is likely to unravel the mechanisms by which these RNAs respond to environmental cues and control cellular phenotypes. Tanwar et al. further elaborate on the role of non-coding RNAs in the various micro- and macro-vascular complications of diabetes, a leading cause of disease associated morbidity and mortality. Their review focuses on long non-coding RNAs (lncRNAs), which have emerged as an important class of epigenetic regulators. The authors discuss the mechanisms by which lncRNA dysregulation contributes to inflammation and vascular complications of diabetes across multiple tissues, and highlight the growing promise of lncRNAs as therapeutics targets.

Environmental cues also affect the epigenome of non-beta cell tissues that control glucose homeostasis. Insulin resistance (IR) is associated with obesity and a variety of metabolic dysfunction phenotypes. Continued IR often progresses to Type 2 diabetes, and is also emerging as a significant factor in poor type 1 diabetes control. Maude et al. focus on the epigenetic basis of hepatic IR, and illustrates that both DNA methylation and histone marks are disrupted in the IR liver at genomic regions relevant to insulin sensitivity and liver function. They further discuss the contribution of short- and long-term epigenetic changes in liver IR and highlight that chronic exposure to adverse stimuli can cause persistent epigenetic dysregulation. Such adverse exposures include parental, *in utero*, or postnatal metabolic challenges, thereby emphasizing the importance of developmental epigenetic patterning in postnatal metabolic health. Andrarde et al. provide a systematic review of available epigenomic data comparing adipose tissue in metabolically healthy- *vs*. unhealthy- obesity (MHO vs MUHO). Their metanalysis identifies important DNA methylation differences in MHO vs MUHO, enriched in loci related to ERK1/2 and small GTPase signaling, as well as vesicular trafficking. This analysis suggests that DNA methylation patterns can potentially distinguish MHO and MUHO, and provides novel insights into why some individuals with obesity may be less susceptible to dysglycemia.

Several common themes emerge from these articles. First, epigenetic dysregulation is central to tissue dysfunction in the pathogenesis of diabetes and associated complications. Second, epigenetic patterning during fetal life is important for postnatal tissue function and its disruption increases diabetes risk in the offspring. Finally, nutrient quality and the timing of nutrient access governs cellular epigenetic programs, affirming the intimate link between metabolism and epigenome. This motif is witnessed during development where nutritional changes direct the epigenetic programs underlying tissue maturation, and in the disease setting, where hyperglycemia serves as a trigger for epigenetic dysregulation and tissue dysfunction. Collectively, this points to common mechanisms that shape the epigenetic response to metabolic changes across tissues, an area of intense investigation that will shape the field in coming years.

## Author Contributions

This editorial was written by SD and revised and approved by HEA, AM-S, and SG. All authors contributed to preparation of this manuscript and approved the final version.

## Conflict of Interest

The authors declare that the research was conducted in the absence of any commercial or financial relationships that could be construed as a potential conflict of interest.

## Publisher’s Note

All claims expressed in this article are solely those of the authors and do not necessarily represent those of their affiliated organizations, or those of the publisher, the editors and the reviewers. Any product that may be evaluated in this article, or claim that may be made by its manufacturer, is not guaranteed or endorsed by the publisher.

